# Advancing implementation science and policy on HIV/AIDS: outcomes from the fourth edition of the Cameroon HIV operational research forum (CAM-HERO) 2024

**DOI:** 10.11604/pamj.2026.53.61.49715

**Published:** 2026-02-05

**Authors:** Gabriel Tchatchouang Mabou, Peter Vanes Ebasone, Nadia Adjoa Sam-Agudu, Patrice Tchendjou, Chibueze Adirieje, Boris Tchounga, Marie Solange Ndom, Joseph Fokam, Rogers Ajeh, Eveline Mboh Khan, Emile Nforbih Shu, Edouard Tshimwanga Katayi, Pascal Nji Atanga, Marc Lionel Ngamani, Clarisse Lengouh, Lorraine Guedem Nekame, Tatiana Djikeussi, Gilbert Tene, Collins Chenwi, Ezechiel Ngoufack Jagni Semengue, Eugene Chiabi, Judith Nasah, Jordanne Ching, Elodie Ngo-Malabo, Emmanuel Nshom, Albert Franck Zeh Meka, Boris Youngui Tchakounte, Charlotte Wenze Ayima, Florence Tumasang, Rogacien Kana, Alex Durand Nka, Francis Ateba Ndongo, Felicite Naah Tabala, Yves Awono Noah, Justin Ndie, Paul Nembo Ngu, Alice Ketchaji, Sandrine Talla, Martine Renée Edakedi, Raissa Banboye, Hannah Kathleen Nzeusseu, Luma Ngonga, Pius Tih Muffih, Anne Cecile Zoung-Kanyi Bisseck, Anastase Dzudie

**Affiliations:** 1 Clinical Research Education, Networking and Consultancy, Yaounde, Cameroon,; 2 Global Pediatrics Program and Division of Pediatric Infectious Diseases, University of Minnesota Medical School, Minneapolis, Minnesota, United States of America,; 3 International Research Center of Excellence, Institute of Human Virology Nigeria, Abuja, Nigeria,; 4 Department of Paediatrics and Child Health, School of Medical Sciences, University of Cape Coast, Cape Coast, Ghana,; 5 The Central and West Africa Implementation Science Alliance (CAWISA), Abuja, Nigeria,; 6 Elizabeth Glaser Pediatric AIDS Foundation (EGPAF), Yaounde, Cameroon,; 7 Douala Laquintinie Hospital, Douala, Cameroon,; 8 Faculty of Medicine and Pharmaceutical Sciences, University of Douala, Douala, Cameroon,; 9 Faculty of Health Sciences, University of Buea, Buea, Cameroon,; 10 National AIDS Control Committee, Ministry of Public Health, Yaounde, Cameroon,; 11 The Global Fund to Fight AIDS, Tuberculosis and Malaria, Ministry of Public Health, Yaounde, Cameroon,; 12 Cameroon Baptist Convention Health Services (CBCHS), Bamenda, Cameroon,; 13 Chantal BIYA International Reference Centre for research on HIV/AIDS prevention and management (CIRCB), Yaounde, Cameroon,; 14 Douala General Hospital, Douala, Cameroon,; 15 Division of Health Operational Research (DROS), Ministry of Public Health, Yaounde, Cameroon,; 16 Division of Epidemic and Pandemic Disease Control (DLMEP), Ministry of Public Health, Yaounde, Cameroon,; 17 Littoral Regional Ethics Committee for Human Health Research, Douala, Cameroon,; 18 Faculty of Medicine and Biomedical Sciences, University of Yaoundé I, Yaoundé, Cameroon,; 19 Department of Internal Medicine and Subspecialities, Douala General Hospital, Douala, Cameroon,; 20 Lown Scholars Program, Department of Global Health and Population, Harvard T.H. Chan School of Public Health, Boston, United States of America

**Keywords:** HIV, research, implementation science, Cameroon, meeting report

## Abstract

Cameroon is dedicated to controlling the HIV epidemic through a coordinated effort led by the Ministry of Public Health and its partners. The fourth edition of the Cameroon HIV Operational Research Forum (CAM-HERO) took place in Douala from December 5-7, 2024, under the theme “Implementation Science and Policy on HIV/AIDS”. The conference brought together local and international researchers, clinicians, and regulatory authorities to: i) disseminate HIV research findings and policies; ii) foster operational research collaboration; iii) build research capacity through training in implementation science; and iv) discuss evidence-based strategies to address key challenges in the national HIV response strategy. A total of 12 oral presentations, 6 poster presentations, and 3 late-breaking abstracts were selected for presentation after a rigorous review process. Key activities included implementation of science training, presentations of selected abstracts, and awards for the best abstract and poster presentations. The conference ended with strong recommendations, including the need to include children and adolescents as participants in the next Cameroon Population-Based HIV Impact Assessment (CAMPHIA). This key recommendation arose because the ongoing CAMPHIA 2024, a cross-sectional household-based, nationally representative survey that will assess the progress of key HIV-related health indicators and describe key HIV-related risk behaviours, gaps and barriers to access HIV care and treatment services include only participants of 15 years and above. This exclusion represents a missed opportunity for HIV research in children.

## Conference proceedings

### Introduction

The Cameroon HIV/AIDS Operational Research Forum (CAM-HERO) has served as a key platform for fostering collaboration among local and international stakeholders, including policymakers, regulatory bodies such as ethics committees, clinicians, and research partners involved in Cameroon´s national HIV response. Since its inception in 2020 [1], CAM-HERO´s goal has been to accelerate the achievement of the UNAIDS 95-95-95 targets in the country by providing an avenue for evidence-based discussions, capacity building, and operational research collaboration, ensuring that research findings effectively translate into policies and programs. Despite significant progress, Cameroon´s HIV response continues to face challenges, particularly in the domains of pediatric HIV care, integration of Communicable and Non-Communicable Diseases (NCDs) control and program sustainability. These challenges were central to the discussions at the fourth edition of CAM-HERO, held from December 5 - 7, 2024, at Hotel la Falaise Bonanjo, in Douala. The forum brought together government authorities, local and international researchers, clinicians, and representatives from key organizations under the leadership of the National AIDS Control Committee (NACC) and the Division of Health Operations Research (DROS) of the Cameroon Ministry of Public Health. CAM-HERO 2024 was built on the successes of previous editions, which have contributed to the development of Cameroon´s National HIV Research Priorities and informed policy decisions [2]. This year´s forum emphasized integrating implementation science into the HIV response, with a particular focus on optimizing pediatric HIV care and addressing barriers to achieving the 95-95-95 targets. The forum featured a three-day program, beginning with a training session on implementation science on the first day, followed by scientific sessions on the second and third days, which included abstract presentations, plenary discussions, and panel sessions. A total of 91 abstracts were submitted (previously 80 in 2021 and 65 in 2022), reflecting the growing interest and commitment to HIV research. After a rigorous review process, 12 abstracts were selected for oral presentation, 6 for poster presentations, and 3 as late-breaking abstracts. These presentations covered a wide range of topics, from molecular epidemiology and treatment monitoring to HIV prevention and community engagement. By fostering collaboration and capacity building, CAM-HERO continues to serve as a cornerstone for advancing HIV research and policy in Cameroon, ultimately contributing to the global goal of ending the HIV epidemic by 2030.

### Day 1: December 5, 2024

**Figure 1:**
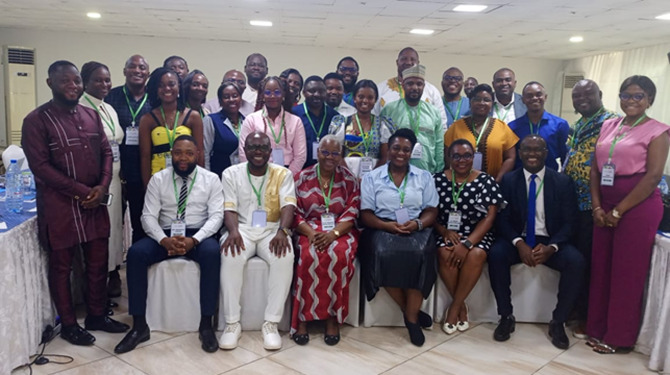
family picture on the first day of the implementation science training

The 4^th^ edition of CAM-HERO commenced with a dedicated training session on Implementation Science, built on training on Research Methodology and Ethics in previous editions [3,4]. This emphasis was in recognition of the growing need for implementation science expertise in Cameroon´s HIV response. The sessions were chaired by Prof. Anne Bissek of the *Division de la Recherche Opérationnelle en Santé/Ministère de la Santé Publique (DROS/MINSANTE)* and facilitated by Prof. Nadia Adjoa Sam-Agudu and Chibueze Adirieje of the Central and West Africa Implementation Science Alliance (CAWISA), Dr. Patrice Tchendjou of Elizabeth Glaser Pediatric AIDS Foundation (EGPAF) and Prof. Anastase Dzudie of Clinical Research Education Network and Consultancy - International epidemiologic databases to evaluate AIDS (CRENC-IeDEA) (Figure 1). Before the training sessions, participants provided information on their current knowledge and expertise in implementation science. The training was divided into four sessions, each focusing on key aspects of implementation science.

**Session 1: core principles of implementation science (IS):** the session aimed at educating attendees on the fundamentals of implementation science. Prof. Sam-Agudu covered the definitions and key terms used in implementation science, implementation research, and implementation practice. She also presented on evidence-based interventions (EBIs), implementation outcomes, effectiveness outcomes, and implementation strategies. A key focus was on developing a research question using the PICOT (Population, Intervention, Comparison, Outcome, Time) model in implementation science. The session emphasized the importance of understanding the use of keywords when establishing an implementation research protocol, as this ensures clarity and alignment with implementation science principles.

**Session 2: theories, frameworks, and models in implementation science:** in this session, Prof. Sam-Agudu introduced the various theories, frameworks, and models (TFM) that form the foundation of implementation science. She emphasized that a protocol lacking these elements is considered an intervention research protocol, not an implementation science protocol. The choice of the appropriate TFM depends on several factors, including attitude, knowledge, training, language, ease of use, evidence, complexity, purpose (outcome), and resources (environmental influence). This session provided participants with the tools to select and apply TFMs effectively in their research projects.

**Session 3: stakeholder, community engagement, and case examples:** in this session, Prof. Sam-Agudu discussed the key principles of stakeholder engagement and outlined the levels of engagement: i) Inform, ii) Consult, iii) Involve, iv) Collaborate, and v) Empower. She also highlighted the 5Ps encountered in implementation research: i) Population, ii) Public, iii) Providers, iv) Payers, and v) Policymakers. The session stressed the importance of engaging the community and stakeholders in implementation research projects to maximize the impact of the outcomes. Real-world case examples were shared to illustrate how effective engagement can lead to successful implementation of evidence-based interventions.

**Session 4: practical session, presentations and discussions:** the final session was a practical workshop where participants were divided into 5 groups. Each group was tasked with the following: i) Describe a health issue; ii) Identify the study population; iii) Identify and formulate EBIs and comparison groups (if any); iv) Formulate a research question using the PICOT model, and v) Consider and select appropriate implementation strategies to address the issue. The groups presented their work, fostering lively discussions and peer feedback. This hands-on approach allowed participants to apply the concepts learned during the training to real-world scenarios, reinforcing their understanding of implementation science principles. At the end of the training, participants took a post-test to assess their understanding of the material covered. Certificates of completion were awarded to participants, recognizing their participation and newly acquired skills in implementation science (Figure 1).

### Day 2: December 6, 2024

**Figure 2:**
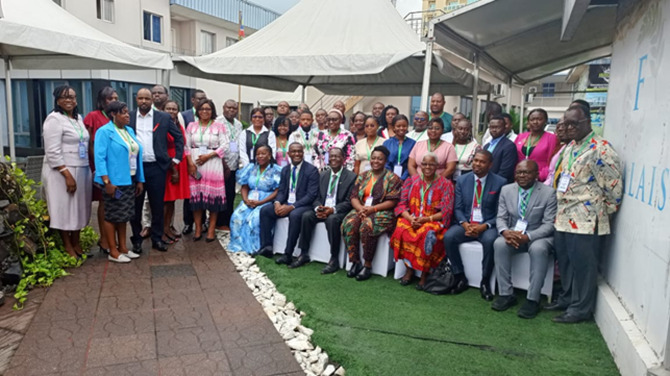
family picture during the second day of the conference

The second day of CAM-HERO 2024 featured abstract sessions and plenary discussions, chaired by Prof. Bissek, Prof. Sam-Agudu, Prof. Pius Tih (Cameroon Baptist Convention Health Service (CBCHS)), Dr. Albert Zeh Meka (National AIDS Control Committee/Ministry of Public Health (CNLS/MINSANTE)), Dr. Patrice Tchendjou (Elizabeth Glaser Pediatric AIDS Foundation (EGPAF)), Dr. Boris Tchounga (EGPAF), Dr. Edouard Tshimwanga (CBCHS), and Dr. Pascal Atanga (CBCHS) (Figure 2). Prof. Bissek officially opened the event, recognizing the significant efforts already made and emphasizing the importance of CAM-HERO as a platform for open engagement between policymakers, program implementers and researchers. Dr. Patrice Tchendjou, Prof. Pius Tih, and Prof. Anastase Dzudie (CRENC-IeDEA) also offered words of appreciation, reiterating their ongoing support and collaboration with the CAM-HERO Consortium. It was also an opportunity to officially introduce Prof. Nadia Adjoa Sam-Agudu and CAWISA as a new strategic partner. CAWISA´s involvement was highlighted as a key step towards strengthening the application of implementation science methods in research and public health practice in the region. On this day, the oral presentation sessions were organized into three main themes.

**Basic science and innovations in diagnostics:** i) *molecular epidemiology of human papillomavirus circulating in African countries according to HIV status: a systematic review, and meta-analysis*. Presented by Moko Fotso Larissa Gaelle, this study explored the association between different HPV genotypes and HIV infection in Africa. The findings highlighted HPV's wide genotypic diversity calling for standard molecular epidemiology investigations to select effective diagnostic methods, optimize clinical management and tailor HPV vaccination policies to locally prevalent genotypes. ii) *A field evaluation of the performance of the duo test HIV/Syphilis SD BIOLINE in Cameroon* by Jean Pierre Yves Awono Noah. This study evaluated the performance of the SD BIOLINE HIV/Syphilis Duo Test in field settings in Cameroon. The results demonstrated that the duo test could lead to improvements in the overall management of HIV and syphilis in the country.

**Clinical science and treatment monitoring:** i) *mental health troubles among Cameroonian adolescents perinatally infected with human immunodeficiency virus* by Jean Pierre Yves Awono Noah. This study examined the prevalence and factors associated with depression in HIV-infected adolescents undergoing antiretroviral treatment in a Cameroonian referral hospital. The study found that increased symptoms of depression, anxiety, and low self-esteem were prevalent among Cameroonian adolescents with perinatal HIV infection. ii) *Predictive efficacy of dual therapy (DT) combining integrase strand transfer inhibitors (INSTIs) with second-generation non-nucleoside reverse transcriptase inhibitors (2^nd^-Gen-NNRTIs) following HIV-1 treatment failure in Cameroon* by Roland Ulrich Wome. This study aimed to assess the predictive efficacy of 2^nd^-Gen-INSTI and 2^nd^-Gen-NNRTI DT-based DT among people living with HIV (PLHIV) failing antiretroviral therapy (ART) in Cameroon through HIV genotypic resistance testing. The findings underscored that INSTIs- and 2^nd^-Gen-NNRTIs-based DT should be reserved for patients with no resistance-associated mutations (RAMs) to NNRTIs and a viral load of less than 50 copies/ml. iii) *Three-year outcome after genotyping-guided switch in HIV multi-drug-resistant patients in Cameroon: evidence-based strategies for achieving epidemic control in low-and middle-income countries* by Magnilack Kuete Chanelle. The study was conducted to evaluate the virological response of people living with HIV following drug-resistant mutations profiling guided treatment switch. Overall, the results showed a good virological response after a genotypic-guided treatment change. These findings align with the national guideline for HIV treatment, revealing: for clients failing 1^st^-line ART: switch to a standard PI/r-based 2^nd^-line ART, and for clients failing 2^nd^-line or PI-based ART: recommend GRT for optimal ART selection. iv) *Evaluation de la charge virale chez les enfants et adolescents infectés par le VIH sous trithérapie à base de dolutegravir à Douala* by Ange Vanelle Pamegni. It was conducted to assess virological suppression in children and adolescents on DTG-based triple therapy. From this study, 93.3% of participants had an undetectable viral load (VL) close to the UNAIDS 3^rd^ target of 95% with parental serostatus being an associated factor with VL suppression.

**HIV prevention and implementation science:** *determinants of HIV retesting among people living with HIV: a cross-sectional study in the North West* Region by Eugene Chiabi. The study aimed to assess the reasons and factors associated with HIV retesting among people living with HIV. The findings showed that retesting is more likely among younger PLHIV and those living in urban settings. Targeting young. ii) *Community engagement of pregnant women in the north region of Cameroon: influence of knowledge toward the prevention of mother-to-child transmission of HIV (PMTCT)* by Jean Pierre Yves Awono Noah. The aim of this study was to assess the influence of knowledge on the community involvement of pregnant and breastfeeding women in the prevention of vertical transmission (mother-to-child transmission (MTCT)) of HIV in the northern region of Cameroon. This study revealed a low level of knowledge and community involvement in HIV PMTCT among women attending antenatal and postnatal consultations. This finding suggests that there is a need to improve knowledge of PMTCT among pregnant and breastfeeding women to increase their involvement and use of PMTCT services. iii) *Acceptability of pre-exposure prophylaxis among adolescents and young men who have sex with men and sex workers in Cameroon: a contribution to preventing HIV infection* by Ndié Justin. The objective of this study was to assess the acceptability of PrEP in the study population. The findings showed that the implementation of PrEP among key populations in Cameroon has been effective in Community-Based Organisations (CBOs) since 2019. The acceptability of PrEP among adolescents and young people in key populations (MSM and SW) in this study was 76.4%. iv) *Menstar approach: an integrated community strategy to reach men with HIV testing services in the Northwest Region* by Eugene Chiabi. The study presented an integrated community-based approach to increase the uptake of HIV clinical services by men. It showed that an integrated package of services to reach men in the community is a feasible and effective approach to increased uptake of HIV testing services among males.

**Plenary sessions:** the plenary sessions on day 2 were chaired by Prof. Bissek, Prof. Tih, Dr. Tchendjou, and Dr. Florence Tumasang (CBCHB). These sessions provided a high-level platform for in-depth discussions on sustainability in the HIV response, pediatric HIV care, and the integration of implementation science into national HIV strategies. Dr. Albert Zeh Meka (NACC/MINSANTE) presented on “leveraging implementation science for HIV sustainability: optimizing program outcomes through evidence-based approaches.” He outlined the critical role of implementation science in ensuring the long-term sustainability of HIV interventions, particularly as Cameroon transitions to locally led HIV programs under the Universal Health Coverage (UHC). Some of the priority areas were eliminating the vertical transmission of HIV, improving HIV care and treatment for children, and optimizing the national strategy for HIV screening and diagnosis. This was followed by Dr. Talla Sandrine´s (EGPAF) talk on “Challenges in PMTCT programs: barriers and strategies”. She identified key bottlenecks in PMTCT program implementation, including stigma and discrimination, gender inequalities, late maternal diagnosis, poor retention in care, insufficient integration of PMTCT with other maternal services, and limited access to early infant diagnosis (EID). She further proposed strategies to enhance service delivery, such as universal health coverage (UHC) as a form of economic support, task-shifting, community engagement, and improved data monitoring systems for appointment reminders and adherence support. Dr. Alice Ketchaji (DLMEP/MINSANTE) then presented on the “pediatric surge project preliminary findings.” which focused on scaling up pediatric HIV case-finding and treatment in Cameroon. Her presentation highlighted achievements in increasing pediatric HIV testing coverage and ART initiation while also identifying gaps in linkage to care and long-term treatment retention. For these preliminary results till November 2024, 68,066 children less than 15 years old were identified and benefited from HIV testing, 735 were tested positive for HIV and all of them were enrolled in ART care. The project helped to identify that 22.2% of HIV-positive cases found were aged 0-14 years, while 30.2 were aged 0-19 years. Overall, 3,774 people living with HIV/AIDS were impacted through initiation or re-initiation of ART. Dr. Albert Zeh Meka (NACC/MINSANTE) concluded the plenary with another comprehensive presentation on “status of pediatric HIV response in Cameroon: an overview of current achievements and Gaps.” He provided a data-driven analysis of pediatric HIV trends in Cameroon, including ART coverage, viral suppression rates, and service delivery challenges. His discussion reinforced the urgent need for continued investment in pediatric HIV research and implementation science to optimize treatment outcomes for children living with HIV. Discussions led the CAM-HERO consortium (CAWISA, CBCHS, CRENC, EGPAF, and NACC) to recognize the urgent need to co-write an implementation science-focused grant to leverage their collaborative strengths.

**Late-breaking abstracts:** important, breaking, and high-quality abstracts were reserved for this special segment of the event. The two late-breaking abstracts presented were: i) The CAMPHIA 2024 (Cameroon Population-Based HIV Impact Assessment) by Dr. Eveline Mboh Khan (CBCHS). This nationwide household survey aims to assess Cameroon´s progress toward achieving the UNAIDS 95-95-95 targets by estimating national and regional HIV incidence and prevalence. It will also examine HIV-related risk behaviors, knowledge gaps, and barriers to accessing HIV services. The findings from CAMPHIA will guide national HIV policy adjustments, inform resource allocation, and shape future research priorities [2]. ii) Biological monitoring of people living with HIV at the Douala General Hospital approved treatment center in Cameroon from January to December 2023 by Dr. Elodie Teclaire Ngo-Malabo who presented the treatment center capacity of the Douala General Hospital with an active cohort of 2,075 patients. Patients aged 50 years and above represented 49.5% of the cohort. The session concluded with closing remarks from Dr. Tchendjou and Prof. Bissek.

### Day 3: December 7^th^, 2024

The final day of the CAM-HERO 2024 featured plenary sessions, poster presentations, and the CAM-HERO Awards of Excellence.

**Plenary sessions:** the day commenced with plenary sessions chaired by Prof. Bissek and Prof. Tih. These sessions aimed to explore the intersection of communicable and non-communicable diseases in HIV care, and important ethical considerations in research within the Cameroon context. Dr. Boris Tchounga (EGPAF) opened the session with a presentation on “Triple Elimination of HIV, Syphilis, and Hepatitis B: Synergistic Approaches, Especially Among Pregnant Women and Vulnerable Populations.” He emphasized the importance of harmonized prevention strategies and the need for integrated screening, diagnosis, and treatment approaches to improve health outcomes in these populations. Following this, Prof. Anastase Dzudie presented on “Burden of NCDs in HIV Patients: Research on the Intersection of HIV and NCDs, and Models for Integrated Care.” His presentation highlighted the increasing prevalence of hypertension, diabetes, and cardiovascular diseases in people living with HIV (PLHIV) and the urgent need for integrated care models to ensure holistic management of these comorbidities. The second plenary session of the day focused on ethics, law, and regulatory challenges in HIV research in Cameroon. The discussions were structured around the evolving legal landscape for research participant protection and strategies to expedite ethical and administrative approvals for research projects. Mr. Paul Ngu (DROS/MINSANTE) presented on “Research participant protection: the implication of the new Research Law” and “The Reinforcement of participant protection by the law: sanctions”. The law was presented with the goal of informing researchers about its provisions regarding human research and participant protection. He outlined the key provisions of Cameroon´s updated research regulations, emphasizing informed consent, data protection, and participant rights. He also discussed sanctions for non-compliance and the need for increased awareness among researchers regarding ethical obligations. Discussions were supplemented with key contributions from Dr. Martine Renée Edakedi and Prof. Dzudie (Regional Ethics-Littoral), and Prof. Bissek (DROS) who shared further experiences. Still in this session, Gabriel Mabou (CRENC-IeDEA) and Lorraine Guedem (EGPAF) presented “The Researcher´s Reflection and Expectation - Insights into researchers' perspectives on study initiation timelines and expectations from the review process”. This segment explored findings from a pre-conference survey that assessed researchers´ perspectives on ethical review timelines, approval delays, and administrative bottlenecks. Overall, this session reviewed and discussed challenges in the ethical review process, including delays in administrative approvals. Common reasons for application rejections, such as incomplete documentation and lack of investigator qualifications. Recommendations for streamlining the ethical clearance process were made including the creation of a centralized digital platform for protocol submission and tracking.

**Poster abstract presentations:** the final poster abstract session was moderated by Dr. Boris Tchounga (EGPAF), Dr. Tshimwanga Edouard (CBCHS), and Dr. Peter Ebasone (CRENC-IeDEA). The abstracts were: i) Low prevalence of HIV in Northern Cameroon: contribution of some AIDS restriction genes and potential implications for gene therapy by Djataou Patrice. ii) Association between Mental Disorders with detectable viral load and poor adherence to antiretroviral therapy among adolescents infected with human immunodeficiency virus on follow-up at Chantal Biya Foundation, Cameroon by Francis Ateba Ndongo. iii) A community-based peer-facilitated psychological and social support model to improve retention in care among Cameroonian adolescents perinatally infected with human immunodeficiency virus: a randomized controlled trial by Jean Pierre Yves Awono Noah. iv) Effects of systematic HIV testing at antenatal clinics and retesting for verification on case identification and testing yield in the West, Southwest and Northwest Regions of Cameroon by Nshom Emanuel Mboh. v) Perception of key stakeholders on the implementation and feasibility of combined HIV-syphilis (SD Bioline HIV/Syphilis Duo) test in pregnant women in Cameroon by Jean Pierre Yves Awono Noah and; vi) Prevalence and Factors Associated with Menopause Among Adult Women Living with HIV in Cameroon by Judith Nasah Lainsi. These poster presentations reinforced the importance of implementation science, community-based interventions, and novel diagnostic approaches in shaping Cameroon´s HIV research landscape.

**CAM-HERO Awards of Excellence:** the day was crowned with the customary CAM-HERO Awards. It was presided over by Prof. Bissek, Director of the DROS/MINSANTE, on behalf of the Ministry of Public Health. Prof. Bissek delivered an inspiring speech, congratulating the CAM-HERO organizers and attendees for their commitment to advancing HIV/AIDS research in Cameroon, and for the successful introduction of implementation Science training on day one. All participants received a Certificate of Participation, while distinguished abstract presenters received special awards. The award winners included: Best Oral Abstract Presentation - Jean Pierre Yves Awono Noah. Second Place Oral Abstract Presentation - Eugene Chiabi and Best Poster Presentation - Francis Ateba Ndongo.

**Key points of the meeting:** the discussions at CAM-HERO 2024 highlighted several components for the future of HIV policy and research in Cameroon, focusing on sustainability, pediatric care, and regulatory evolution. The key points of the meeting are summarized in Table 1.

**Table 1:** summary of the key points of the meeting

Meeting component	Summary
Meeting theme and objective	Fourth edition of the Cameroon HIV Operational Research Forum (CAM-HERO) was held in Douala from 5^th^ to 7^th^ December 2024 under the theme “Implementation Science and Policy on HIV/AIDS,” aimed at disseminating research findings, strengthening collaboration, building research capacity, and supporting evidence-based HIV policies.
Participants	National and international researchers, clinicians, policymakers, regulatory authorities, ethics committees, and implementing partners are involved in Cameroon’s HIV response.
Implementation science training	One-day training focused on core principles of implementation science, theories, frameworks and models, stakeholder and community engagement, and practical group exercises using the PICOT model.
Abstract submissions and selection	A total of 91 abstracts were submitted; 12 were selected for oral presentations, 6 for poster presentations, and 3 as late-breaking abstracts following a rigorous review process.
Abstract presentations	Oral and poster presentations covered basic science and diagnostics, clinical science and treatment monitoring, HIV prevention, community-based interventions, and implementation science. Late breaking abstract presentations included preliminary findings from CAMPHIA 2024 and biological monitoring data from a major treatment center in Cameroon.
Pediatric HIV focus	Multiple presentations addressed pediatric and adolescent HIV, including viral suppression, PMTCT, mental health, case finding, and treatment outcomes.
Plenary sessions	High-level discussions on sustainability of the HIV response, pediatric HIV care, integration of implementation science, triple elimination of HIV, syphilis and hepatitis B, and the intersection of HIV and non-communicable diseases.
Ethics and regulatory discussions	Sessions addressed Cameroon’s updated research law, participant protection, informed consent, data protection, sanctions for non-compliance, and challenges in ethical and administrative review processes.
Awards and recognition	CAM-HERO Awards of Excellence were presented for best oral and poster abstract presentations, and all participants received certificates of participation.
Key recommendation	Strong recommendation to include children and adolescents in future Cameroon Population-Based HIV Impact Assessment (CAMPHIA) surveys.

## Conclusion

The fourth edition of CAM-HERO successfully upheld its mission of fostering collaboration, strengthening research capacity, and promoting the integration of implementation science into the national HIV/AIDS response in Cameroon. Through engaging plenary sessions, abstract presentations, and high-level panel discussions, the forum provided a unique platform for researchers, policymakers, and stakeholders to share findings, address key challenges, and formulate evidence-based strategies. Key achievements included the incorporation of implementation science training, discussions on optimizing pediatric HIV care, and the reinforcement of ethical and regulatory frameworks for research in Cameroon. The event further highlighted the need for continued investment in research capacity building, improvement of the ethical review process, data standardization, the importance of human subject protection in research, and the development of innovative approaches to improve retention in HIV care and treatment outcomes.
